# Elevated Neopterin Levels Are Associated with Increased Tuberculosis Risk in Rheumatoid Arthritis Patients with QuantiFERON Conversion during Biologic Therapy

**DOI:** 10.1371/journal.pone.0166301

**Published:** 2016-11-18

**Authors:** Der-Yuan Chen, Ju-Pi Li, Yi-Ming Chen, Tsai-Ling Liao, Hsin-Hua Chen, Chia-Wei Hsieh, Yea-Wen Yeh, Joung-Liang Lan

**Affiliations:** 1 Division of Allergy, Immunology and Rheumatology, Department of Medical Education and Research, Taichung Veterans General Hospital, Taichung, Taiwan; 2 Faculty of Medicine, National Yang Ming University, Taipei, Taiwan; 3 Institute of Biomedical Science and Rong Hsing Research Center for Translational Medicine, National Chung Hsing University, Taichung, Taiwan; 4 Institute of Biochemistry, Microbiology and Immunology, Chung Shan Medical University, Taichung, Taiwan; 5 Division of Immunology and Rheumatology, Department of Medicine, China Medical University Hospital, Taichung, Taiwan; 6 College of Medicine, China Medical University, Taichung, Taiwan; Hospital Universitari de Bellvitge, SPAIN

## Abstract

QuantiFERON-TB-Gold (QFT-G) conversion is frequently observed in rheumatoid arthritis (RA) patients receiving biologic therapy. However, there have not been any known biomarkers available for detecting tuberculosis (TB) in QFT-G converters. We aimed to evaluate clinical utility of cytokines/chemokines for detecting TB in patients with QFT-G conversion. Among a total of 227 RA patients who underwent QFT-G assay, 187 QFT-G-negative patients received biologic therapy without isoniazid prophylaxis. QFT-G assay was repeated at week 52 of biologic therapy or at the time of TB diagnosis. Levels of cytokines/chemokines were determined by magnetic bead array or ELISA in QFT-G converters and 12 non-RA patients with TB (non-RA TB). QFT-G conversion was found in 54 (28.9%) of 187 baseline QFT-G-negative patients, of which 7 (13.0%) developed active TB during the one-year follow-up period. Among the examined cytokines/chemokines, non-stimulated and TB-antigen-stimulated neopterin levels were significantly higher in RA patients who developed TB (RA-TB) (median, 24.5pg/ml and 23053pg/ml, respectively) and non-RA TB patients (12.2pg/ml and 9633pg/ml, respectively) compared with QFT-G converters without TB (3.0pg/ml and 2720pg/ml, respectively, both p<0.001). Rising levels of neopterin relative to baseline (non-stimulated levels, 4.4pg/ml vs. 24.5pg/ml; TB-antigen-stimulated levels, 1801pg/ml vs. 23053pg/ml) were observed in QFT-G converters who developed TB. A high proportion (85.7%) of QFT-G converters with high plasma neopterin levels developed TB during the one-year follow-up period. In conclusion, RA patients with QFT-G conversion during the period of biologic therapy should be carefully monitored for elevation of neopterin levels, which is associated with TB risk in QFT-G converters, particularly in TB-endemic areas.

## Introduction

Tuberculosis (TB) remains a major global public health issue. An estimated 9.0 million people developed TB and 1.5 million died from the disease in 2013 [1]. There is a high prevalence of TB in Taiwan, despite the extensive implementation of TB control measures and universal Bacillus Calmette-Guérin (BCG) vaccination [2]. An increased TB prevalence has been reported in rheumatoid arthritis (RA) patients [3], and its risk increased further in those receiving biologic therapy [4–6]. Guidelines have recommended that screening for latent TB infection (LTBI) should be carried out and isoniazid prophylaxis (INHP) be initiated if LTBI exists [7].

Accumulating evidence indicates that QuantiFERON-TB Gold (QFT-G) assays, which detect interferon (IFN)-γ secreted by T-cells stimulated with *M*. *tuberculosis (Mtb)*-specific antigens, offer higher specificity than tuberculin skin test (TST) in detecting LTBI or active TB within a BCG-vaccinated population [8–9]. Therefore, QFT-G assays are preferred when testing for LTBI in BCG-vaccinated subjects [10–11], and replacing TST with QFT-G assays for LTBI detection allowed 16.4% of RA patients to avoid unnecessary prophylactic therapy [12]. However, neither QFT-G assay nor TST allows for discrimination between LTBI and active TB [13].

Recently, Diel *et al*. reported that QFT-G assay was more reliable than TST in identifying TB-contacts who will soon progress to active TB [14], and Machingaidze *et al*. indicated that recent QFT-G conversion was indicative of an eight fold higher risk of developing active TB [15]. We recently also demonstrated that QFT-G conversion strongly indicate the emergence of active TB in patients undergoing anti-TNF-α therapy [16]. However, Hatzara *et al*. demonstrated that no rheumatic patients developed active TB during follow-up in spite of frequent QFT-G conversion [17]. Therefore, there is an urgent need to identify candidate markers for effectively detecting active TB in QFT-G converters during the period of biologic therapy.

Cellular immunity plays a critical role in the response to *Mycobacterial tuberculosis* (*Mtb*) infection, and T cells/macrophages secrete cytokines such as IFN-γ, interleukin (IL)-2, IL-6, IL-17A and TNF-α, and chemokines such as IL-8 and IFN-γ inducible protein-10 kDa (IP-10, CXCL10) to control TB infection [18–21]. Th2 cytokines have been shown to be increased in the plasma of advanced TB patients [22]. Neopterin, a pteridine derivative (pyrazinopyrimidine) that is synthesized by activated macrophages, is considered to be a marker of cellular immune activation [23–24]. Therefore, we hypothesized that certain markers of cell-mediated immunity to *Mtb* infection could be used to detect active TB in RA patients with QFT-G conversion during the period of biologic therapy.

A recent study indicated that the ratio of TB-specific response to mitogen-stimulated responses for IL-2, IL-6, IL-10, IL-13, TNF-α, IFN-γ, monokine induced by IFN-γ (MIG) and IP-10 were useful in discriminating active TB from LTBI [25]. Recent studies also revealed that plasma levels of CXC chemokine receptor 3 (CXCR3) ligands might be useful markers for detecting active TB [26], and combined analysis of cytokines/chemokines in QFT supernatant is useful for distinguishing active TB from latent infection [27]. However, there are no data on candidate cytokines or chemokines for detecting active TB in RA patients with QFT-G conversion during the period of biologic therapy.

In the present study, we investigated: 1) the differences in non-stimulated levels, TB antigens- or mitogen-stimulated levels of cytokines/chemokines between RA QFT-G converters with and without developing active TB; and 2) the change in plasma levels of cytokines/chemokines in RA QFT-G converters during one-year biologic therapy or at the time of TB diagnosis.

## Methods

### Study population

**Fig 1:**
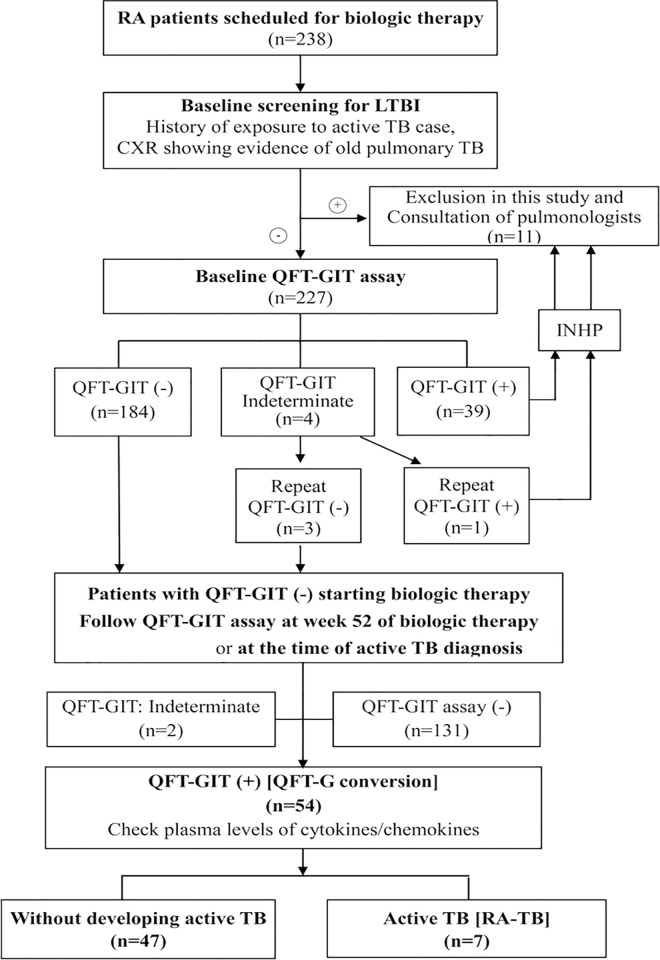
Flow chart shows the distribution of results of QuantiFERON-TB Gold In-Tube (QFT-GIT) assay in 238 patients with rheumatoid arthritis (RA) before and after anti-TNF-α therapy. Seven patients developed active tuberculosis (TB) disease after anti-TNF-α therapy. LTBI: latent TB infection; INHP: isoniazid prophylaxis; QFT-GIT (+): positive QFT-GIT result; QFT-GIT (-): negative QFT-GIT result. QFT-GIT conversions were defined as: 1) baseline IFN-γ <0.35 IU/ml and follow-up IFN-γ≧0.35 IU/ml; and 2) baseline IFN-γ <0.35 IU/ml and follow-up IFN-γ≧0.70 IU/ml.

Two hundred thirty-eight biologic-naïve RA patients [28] scheduled to receive biologic therapy were consecutively enrolled: 112 patients scheduled to receive adalimumab, 66 etanercept, 16 golimumab, 28 abatacept, and 16 tocilizumab, all with concomitant methotrexate (MTX) therapy at a stable dose of 7.5–15 mg weekly. Patients with persistently active disease received biologic therapy at standard doses based on the British Society for Rheumatology guidelines [29]. All patients were evaluated before biologic therapy using a standardized interview, and chest radiographs (CXR). After exclusion of 11 patients with clinically active TB or suspicious active TB from CXR, 227 patients underwent QFT-G In-tube assay before starting biologic therapy. Forty QFT-G-positive patients who were assumed to have LTBI and received INHP before starting biologic therapy were also excluded. A total of 187 baseline QFT-G-negative patients started biologic therapy with concomitant use of methotrexate (MTX) at a stable dose of 7.5–15 mg weekly, and QFT-G assay was repeated at week 52 of biologic therapy or at the time of active TB (Fig 1). Disease activity was assessed by the 28-joint disease activity score (DAS28) [30]. Clinical investigators for RA disease activity were blinded of the results of QFT-G assay and cytokines/chemokines. We also enrolled the other 12 newly diagnosed TB patients without rheumatic diseases (non-RA TB) as disease controls. RA patients were closely and regularly monitored for the development of active TB which was proved by positive culture or pathological findings. The Ethics Committee of Taichung Veterans General Hospital approved this study (C09162) and the written consent from each participant was obtained according to the declaration of Helsinki.

### QFT-G assay for RA patients and non-RA TB patients

QFT-G assay was performed according to the manufacturer’s instructions (Cellestis Ltd., Victoria, Australia). The result of QFT-G assay was defined as positive if IFN-γ level≧0.35 IU/ml in TB-specific antigens-stimulated wells after subtracting the level of the nil well according to the manufacturer’s recommendation. QFT-G conversion was defined as baseline IFN-γ <0.35 IU/ml and follow-up IFN-γ≧0.35 IU/ml [15,31]. Borderline QFT-G conversion was defined if the change of TB antigen-specific IFN-γ level was 0.35–0.70 IU/ml, while non-borderline conversion was defined as the change of IFN-γ level >0.70 IU/ml in the follow-up assays, similar to the previous reports [15,31].

### Measurement of plasma levels of cytokines and chemokines

Levels of IP-10, IFN-γ, TNF-α, IL-2, IL-6, IL-17A, IL-8, IL-10, and IL-13 were determined in plasma samples (nil wells, TB antigen-stimulated wells, and mitogen-stimulated wells) collected in QFT-GIT test using the Milliplex MAP Cytokine/Chemokine Panel assay according to the manufacturer’s instruction (EMD Millipore, Waltham, MA, USA). Neopterin levels were detected using enzyme-linked immunosorbent assay (RE59321, IBL international GmbH, Germany).

### Statistical analysis

Results are presented as mean±SD unless specified otherwise. The nonparametric Kruskal-Wallis test was used for between-group comparison. Only when this test showed significant differences were the exact p values determined using the Mann-Whitney U test. For the comparison of cytokines as well as chemokines before and after biologic therapy during follow-up period, the Wilcoxon signed rank test was employed. The optimal cutoff values of neopterin levels and IFN-γ released levels in QFT-G assay for detecting active TB were determined using receiver-operating characteristic (ROC) curves. *P* values <0.05 were considered statistically significant.

## Results

### QFT-G conversion and TB development during the period of biologic therapy

Of 187 RA patients with baseline QFT-G-negative results, 54 (28.9%) were found to have QFT-G conversion. Among 54 QFT-G converters, 12 were borderline converters, none of whom developed active TB during a one-year follow-up period.

Of 42 RA patients with non-borderline conversion, 7 developed active TB (RA-TB) during the period of biologic therapy: 2 patients developed TB within the first four months of adalimumab therapy, while the other 5 after nine months of therapy with adalimumab (n = 2) or etanercept (n = 3). Among them, 5 (71.4%) had extrapulmonary involvement, including subcutaneous abscess (2/5), pleural, pericardial or peritoneal TB (2/5), and miliary TB (1/5). Of 12 non-RA subjects with active TB (non-RA TB), 5 (41.7%) had extrapulmonary TB, including arthritis (2/5), pleura (2/5), and subcutaneous abscess (1/5).

### Demographic data and clinical characteristics

As illustrated in Table 1, RA-TB patients were older than those without development of active TB (RA non-TB). In addition, a significantly lower proportion of female was observed in non-RA TB patients compared with RA patients with or without developing TB. However, there were no significant differences in the positive rates of rheumatoid factor and anti-cyclic citrullinated peptide antibodies, disease activity, proportion of BCG vaccination, daily dosage of corticosteroid used, or proportion of the used DMARDs between RA-TB and RA non-TB patients.

**Table 1:** Demographic data and laboratory findings of RA quantiFERON converters with and without development of active TB, and non-RA subjects with active TB^#^.

	RA without development of TB (n = 47)	RA with development of TB (n = 7)	Non-RA with active TB (n = 12)
Mean age at study entry, years	55.7 ± 12.8	64.0 ± 7.9*	59.8 ± 21.3
Female proportion	40 (85.1%)	6 (85.7%)	4 (33.3%)*§
RF positivity	32 (68.1%)	4 (57.1%)	NA
Anti-CCP positivity	31 (66.0%)	4 (57.1%)	NA
ESR (mm/1^st^ hour)	42.7 ± 21.5	47.1 ± 18.5	NA
Baseline DAS28	6.11 ± 0.66	6.34 ± 0.57	NA
BCG vaccination	43 (91.5%)	6 (85.7%)	11 (91.7%)
Daily steroid dose (mg)	5.6 ± 2.1	6.8 ± 1.9	NA
Concomitant DMARDs			
Methotrexate	41 (87.2%)	6 (85.7%)	NA
Sulfasalazine	28 (59.6%)	4 (57.1%)	NA
Hydroxychroloquine	24 (51.1%)	4 (57.1%)	NA
Biologics used			
Adalimumab	20 (80.0%)	5 (20.0%)	NA
Etanercept	10 (83.3%)	2 (16.7%)	NA
Golimumab	4 (100%)	0 (0.0%)	NA
Abatacept	9 (100%)	0 (0.0%)	NA
Tocilizumab	4 (100%)	0 (0.0%)	NA
Frequency of comorbidities			
Diabetes mellitus	2 (4.3%)	0 (.0%)	0 (0.0%)
Anemia (<9.0 gm/dl)	10 (21.3%)	2 (28.6%)	1 (8.3%)

#Values are mean ± standard deviation or the number (%) of patients.

RA: rheumatoid arthritis; TB: tuberculosis; RF: rheumatoid factor; Anti-CCP: anti-cyclic citrullinated peptide antibodies; BCG: Bacillus Calmette-Guérin; DAS28: disease activity score for 28-joints; DMARDs: disease-modifying anti-rheumatic drugs; NA: not applicable.

*p<0.005 versus RA patients without development of active TB

^**§**^p<0.05 versus RA patients with development of active TB

### Non-stimulated levels of neopterin, IP-10, IFN-γ, TNF-α, IL-2, IL-6, IL-17A, IL-8, IL-10, and IL-13 in patients with RA-TB, RA non-TB, and non-RA TB

**Fig 2:**
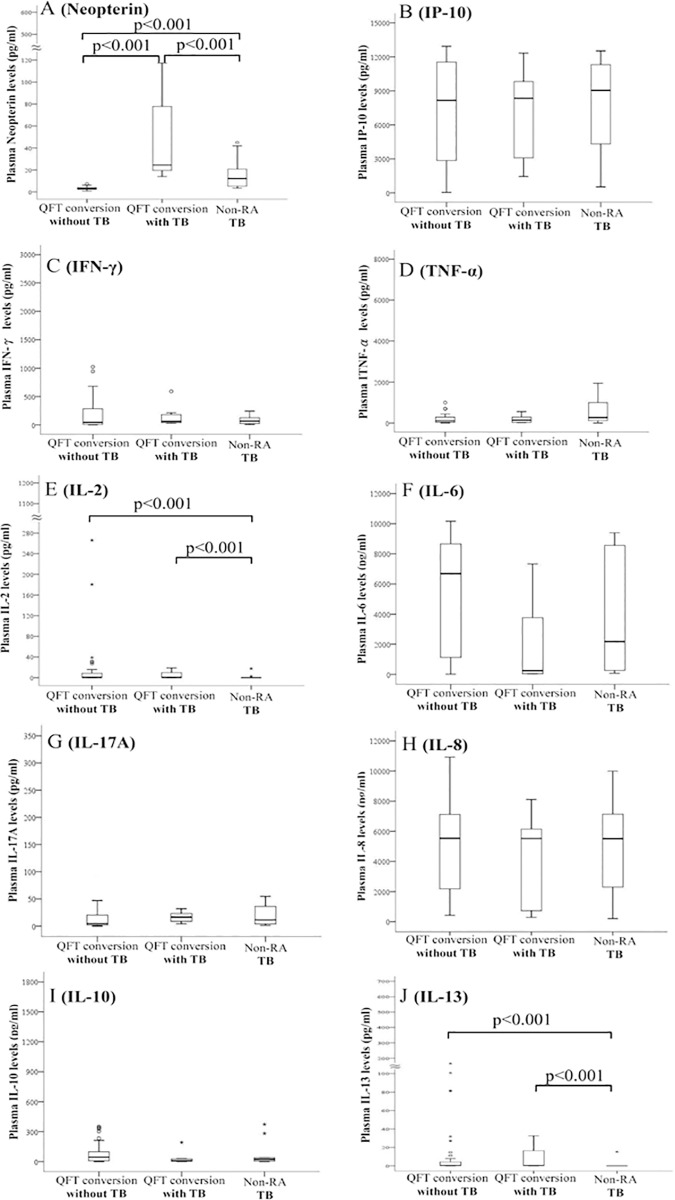
Comparison of non-stimulated levels of cytokines and chemokines at week 52 or at the time of TB diagnosis in RA QFT-G converters with and without developing active TB, and non-RA subjects with TB. RA: rheumatoid arthritis; QFT-G: quantiFERON-TB-Gold assay; IP-10: IFN-γ inducible protein-10 kDa; IFN-γ: interferon-γ; TNF-α: tumor necrosis factor-α; IL: interleukin; Data are presented as box-plot diagrams, with the box encompassing the 25^th^ percentile (lower bar) to the 75^th^ percentile (upper bar). The horizontal line within the box indicates median value respectively for each group.

Significantly higher non-stimulated median levels of neopterin were observed in RA-TB patients (24.5pg/ml) and non-RA TB patients (12.2pg/ml) compared with RA non-TB patients (3.0pg/ml, both p<0.001, Fig 2A). Non-stimulated neopterin levels were also significantly higher in RA-TB patients than in non-RA TB patients (p<0.001, Fig 2A). In the contrast, non-stimulated levels of IL-2 and IL-13 were significantly lower in non-RA TB patients compared with RA-TB patients or RA non-TB patients (Fig 2). There were no significant differences in levels of IP-10, IFN-γ, TNF-α, IL-6, IL-17A, IL-8, or IL-10 between RA converters and non-RA TB patients or between RA-TB patients and RA non-TB patients (Fig 2).

### Stimulated levels of neopterin, IP-10, IFN-γ, TNF-α, IL-2, IL-6, IL-17A, IL-8, IL-10, and IL-13 in in patients with RA-TB, RA non-TB, and non-RA TB

**Fig 3:**
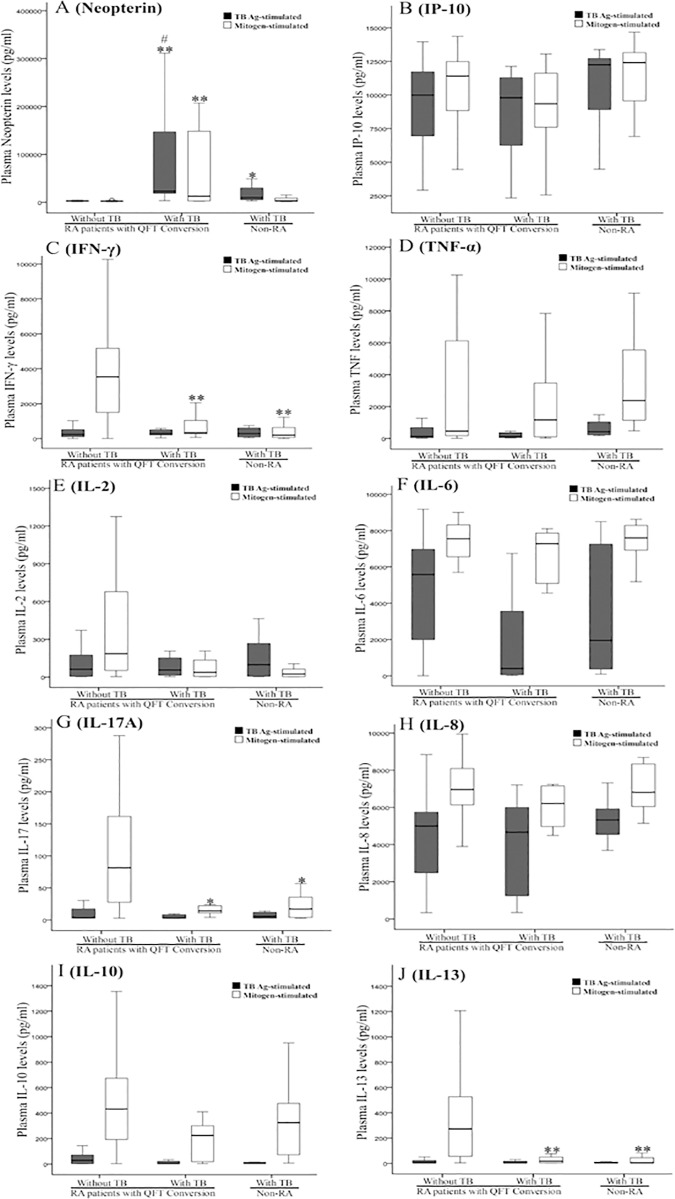
Comparison of stimulated levels of cytokines and chemokines at week 52 or at the time of TB diagnosis in RA QFT-G converters with and without developing active TB, and non-RA subjects with TB. The abbreviations are as described in Fig 2. Data are presented as box-plot diagrams, with the box encompassing the 25^th^ percentile (lower bar) to the 75^th^ percentile (upper bar). The horizontal line within the box indicates median value respectively for each group. *p<0.05, **p<0.001, versus RA patients without development of active TB; #p<0.001, versus non-RA TB patients

TB antigen-stimulated levels of neopterin were significantly higher in RA-TB patients and non-RA TB patients than in RA non-TB patients (Table 2 and Fig 3A). Levels of neopterin were also significantly higher in RA-TB patients than in non-RA TB patients (p<0.001, Fig 3A). However, there were no significant differences in TB antigen-stimulated levels of IP-10, IFN-γ, TNF-α, IL-2, IL-6, IL-17A, IL-8, IL-10, or IL-13 between RA-TB patients and RA non-TB patients or non-RA TB patients (Table 2 and Fig 3B–3I).

**Table 2:** Stimulated levels of cytokines and chemokines in rheumatoid arthritis (RA) quantiFERON converters with and without development of active TB, and non-RA subjects with active TB (non-RA TB).

Levels of cytokines or chemokines	RA without development of active TB (n = 47)		RA with development of active TB (n = 7)		Non-RA TB (n = 12)
	At baseline	At week 52	At baseline	At the time of TB diagnosis	At the time of TB
Neopterin (TB Ag)	2538(1865–2912)	2720(1928–3342)	1801(1683–3704)	23053(16060–244922)** #	9633(4463–31413)*
Neopterin (mitogen)	2371(1741–3228)	2290(1801–2912)	2290(1075–3342)	12642(2538–176814)**	2875(1695–10071)
IP-10 (TB Ag)	5051(2359–8532)	9987(6769–11772)	4043(1725–7877)	9798(5125–11901)	12249(7591–12742)
IP-10 (mitogen)	11111(8685–12141)	11412(8550–12529)	9411(4680–12357)	9349(6968–11825)	12403(9034–13217)
IFN-γ (TB Ag)	42.83(7.24–88.14)	244.58(100.98–533.62)	17.78(2.66–59.52)	280.74(186.11–564.01)	281.32(91.24–680.09)
IFN-γ (mitogen)	1407(574.91–3948)	3533(1432–5271)	238.3(152.6–2021)**	339.11(216.23–1528)**	199.37(54.75–776.89)**
TNF-α (TB Ag)	88.93(8.78–227.18)	134.99(43.38–720.14)	110.14(77.39–176.89)	118.72(45.15–461.84)	420(210.46–1046)
TNF-α (mitogen)	1063(156.89–3769)	457.43(170.61–6255)	1163(210.12–2483)	1165(82.72–4175)	2376(994.18–5798)
IL-2 (TB Ag)	20.54(6.28–76.96)	60.72(4.51–184.24)	8.45(2.86–56.33)	55.54(7.35–205.57)	97.30(5.05–293.31)
IL-2 (mitogen)	288.99(57.12–741.48)	185.39(50.86–691.28)	47.74(25.87–203.29)	37.46(2.86–206.18)	23.21(2.86–68.81)
IL-6 (TB Ag)	580.83(193.55–1858)	5584(1974–7156)	235.61(51.98–1049)	406.69(38.18–6626)	1952(369.21–7528)
IL-6 (mitogen)	7012(5814–7906)	7551(6561–8350)	5741(2036–7236)	7280(4559–7995)	7598(6873–8408)
Il-17A (TB Ag)	3.20(2.83–7.86)	3.52(2.83–18.01)	2.83(2.83–10.39)	3.01(2.83–9.80)	5.50(2.85–13.09)
Il-17A (mitogen)	52.97(19.91–139.5)	81.46(25.88–164.04)	5.81(2.83–25.51)*	14.05(8.93–23.99)*	17.13(3.85–37.60)*
IL-8 (TB Ag)	3867(1686–5589)	5000(2450–5795)	3376(1583–5510)	4669(1217–6138)	5330(4459–6003)
IL-8 (mitogen)	6622(5197–7726)	6968(6126–8162)	6465(6323–7537)	6212(4493–7166)	6815(6006–7345)
IL-10 (TB Ag)	2.89(2.89–21.57)	27.93(2.89–77.57)	3.08(2.89–24.21)	2.89(2.89–33.07)	4.86(2.89–13.78)
IL-10 (mitogen)	249.28(107.02–395.72)	433.10(186.48–675.60)	194.29(113.24–568.91)	223.78(9.79–340.60)	324.65(72.22–492.18)
IL-13 (TB Ag)	3.66(3.66–3.66)	3.66(3.66–24.94)	3.66(3.66–3.66)	3.66(3.66–30.41)	3.66(3.66–12.14)
IL-13 (mitogen)	255.71(32.44–621.52)	272.91(45.91–528.25)	22.17(3.66–339.65)**	17.29(3.66–75.91)**	3.66(3.66–46.45)**

Values are median value (95% confidence interval); TB: tuberculosis; Ag: antigen; IP-10: IFN-γ inducible protein-10 kDa; IFN-γ: interferon-γ; TNF-α: tumor necrosis factor-α; IL: interleukin.

*p<0.05

**p<0.001, vs. RA patients without active TB

#p<0.05, vs. non-RA TB patients

Interestingly, mitogen-stimulated levels of IFN-γ, IL-17A and IL-13 were significantly lower in active TB patients (RA-TB and non-RA TB) compared with RA non-TB patients (Table 2 and Fig 3C, 3G and 3J).

### Correlations between neopterin levels and IFN-γ levels or RA disease activity

Non-stimulated neopterin levels were positively correlated with IFN-γ levels (r = 0.280, p<0.05) and ESR assessed at baseline (r = 0.315, p<0.05), and TB-antigen stimulated neopterin levels were positively correlated with IFN-γ levels (r = 0.322, p<0.05) and DAS28 score assessed at baseline (r = 0.310, p<0.05) in RA patients.

### The difference in neopterin levels between RA patients with borderline and non-borderline conversion of QFT-G assays

Of the 54 QFT-G converters, 12 had borderline conversion. Non-stimulated and TB antigen-stimulated levels of neopterin were lower in patients with borderline QFT-G conversion (median = 2.6pg/ml, interquertile range [IQR] 1.3–3.3pg/ml and 2429pg/ml, IQR 1660-3210pg/ml; respectively) than in those with non-borderline conversion (median = 3.5pg/ml, IQR 2.4–5.7pg/ml, p = 0.167 and 2963pg/ml, IQR 2177-4287pg/ml, p = 0.086; respectively).

### Comparison of plasma neopterin levels and IFN-γ levels in RA QFT-G converters with and without developing active TB

**Fig 4:**
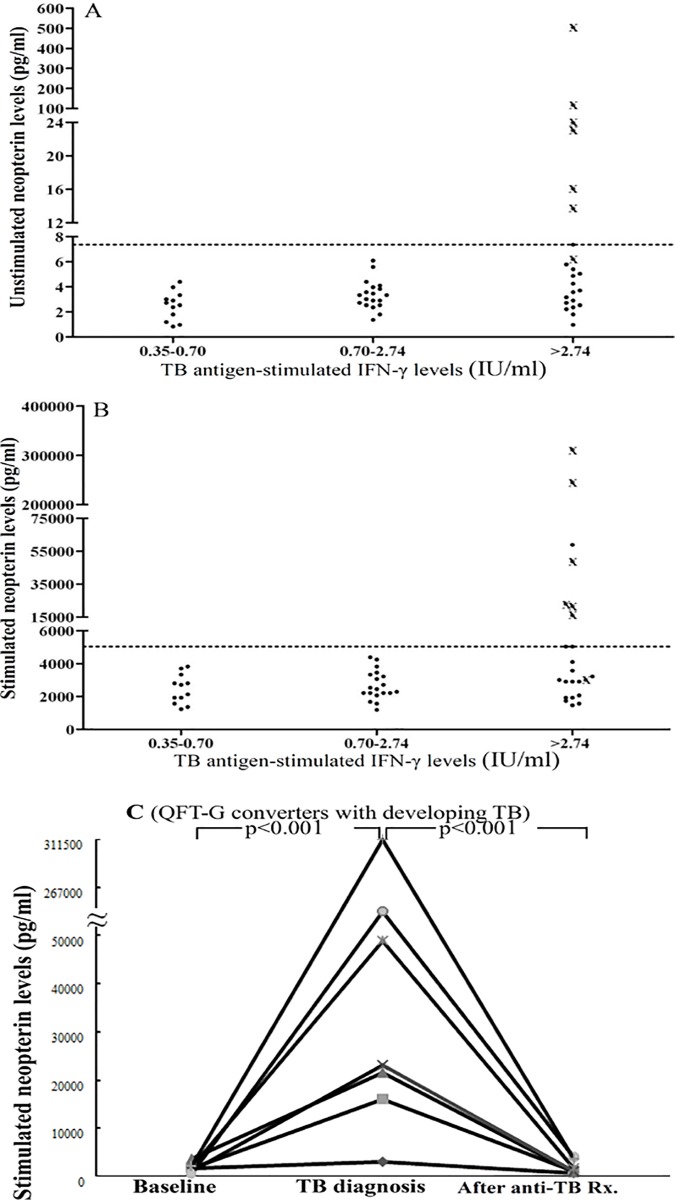
Comparison of plasma levels of non-stimulated (A) and stimulated (B) neopterin and IFN-γ levels in QFT-G assay for 54 RA QFT-G converters with and without developing active TB. Seven QFT-G converters who developed active TB are marked (X). Strong responses equal to or greater than 2.74 IU/ml for the QFT-G assay are determined by receiver-operating characteristic (ROC) curves analysis. The dotted line represents the cutoff value of 7.36pg/ml for non-stimulated neopterin levels and 5045pg/ml for stimulated neopterin levels. (C) The changes in TB-antigen-stimulated neopterin levels in seven QFT-G converters who developed TB during the period of biologic therapy and after the completion of anti-TB therapy. *P*-value was determined by Wilcoxon signed rank test.

Seven (13%) of the 54 RA QFT-G converters had developed active TB (RA-TB). The TB-antigen-stimulated IFN-γ levels in QFT-G assay were all above a cutoff value of 2.74IU/ml in our RA-TB patients (Fig 4). In contrast, 16 QFT-G converters whose IFN-γ released levels were above 2.74 IU/ml did not develop active TB (a specificity of 66.0%). For plasma neopterin levels, six of seven RA-TB patients had high levels of non-stimulated neopterin with a cutoff value of 7.36pg/ml (Fig 4A), and high levels of stimulated neopterin with a cutoff value of 5045pg/ml (Fig 4B). Only one QFT-G converter who did not develop active TB had a high neopterin levels above the cutoff value with a specificity of 85.7%.

### Change in neopterin levels in RA QFT-G converters who developed active TB

As shown in Fig 4C, significantly rising levels of neopterin (TB antigen-stimulated) were observed in 7 RA QFT-G converters who developed TB (median, 1801pg/ml vs. 23053pg/ml, p<0.001), while no significant change in neopterin levels was observed in those without developing TB (median, 2537.6pg/ml vs. 2719.8pg/ml). In addition, TB antigen-stimulated neopterin levels significantly decreased after the completion of anti-TB therapy (median, 23053pg/ml vs. 1000pg/ml, p<0.001, Fig 4C).

## Discussion

Although QFT-G assays have been used to detect active TB, the pooled sensitivity and specificity were at merely 80% and 79% respectively [32]. Therefore, combining the results of QFT-G results with *Mtb*-specific immune markers might improve the diagnostic ability to detect active TB. The present study is the first to evaluate the clinical utility of immune activation markers for detecting active TB in RA QFT-G converters. Among the examined cytokines/chemokines, neopterin levels were significantly higher in 7 RA-TB and 12 non-RA TB patients compared with 47 RA non-TB patients. There was little overlap of the range of neopterin levels between QFT-G converters with and without developing TB. Moreover, a significant rise of neopterin levels in RA-TB patients at the time of TB diagnosis was observed. These results suggest that elevated neopterin levels are associated with TB risk in RA QFT-G converters during biologic therapy.

Similar to a recent study showing frequent conversion of LTBI screening tests in RA patients receiving biologic therapy [17,33], 28.9% of our RA patients had QFT-G conversion during one-year biologic therapy using follow-up IFN-γ≧0.35 IU/ml as the criteria. In contrast to the result of no occurrence of TB observed in treated QFT-G converters in one Greek study [17], 7 (13%) of our untreated converters developed active TB. This discrepancy might be due to 1) the differences in the characteristics of the enrolled patients (with versus without INHP); and 2) the difference in the prevalence of active TB (49.4/100,000 Taiwan populations versus 5.0/100,000 Greece populations). However, the rate of progression to active TB in our study was similar to that at 12.9% (19 of 147) among TB-exposed subjects who had positive QFT-G results without INHP in another study [14], resonating with the findings that recent QFT-G converters had an eight fold higher risk of active TB development compared with non-converters [15].

It is interesting that TB Ag-stimulated IFN-γ levels in QFT-G assay were all above 2.74 IU/ml in our RA-TB patients, similar to previous findings that 16 of 19 contacts with progression to TB had strong IFN-γ responses >3.5 IU/ml in QFT-G assay [14]. However, the specificity was low (66.0%) if IFN-γlevel≧2.74 IU/ml was used to identify TB development in our QFT-G converters. Another recent report similarly revealed a poor specificity (64.9%) of QFT-G assay in detecting active TB in a TB-endemic area [34]. Therefore, QFT-G assay alone as a diagnostic tool is not adequate for identifying active TB.

Neopterin is produced by activated macrophages in response to IFN-γ, and therefore is a marker of cell-mediated immunity in inflammatory diseases, such as TB and RA [23–24,35]. The role of cell-mediated inflammation in neopterin production was supported by the positive correlations between neopterin levels and IFN-γ levels, and between neopterin levels and disease activity in our RA patients. To explore the RA-related elevation of neopterin levels [35], we also enrolled non-RA subjects with active TB as a control group. Neopterin levels were significantly higher in our RA-TB patients compared with non-RA TB patients, suggesting a further increase of neopterin levels driven synergistically by RA-related inflammation [35].

Among RA QFT-G converters, significantly increased neopterin levels relative to baseline were observed in those who developed active TB (Fig 4C), supporting other studies which showed high neopterin levels in active TB patients [23–24,36–37], and in TB-exposed health-care workers who developed TB [38]. Similar to another recent study [39], neopterin levels significantly decreased after the completion of anti-TB therapy in our RA-TB patients. However, further validation of the clinical utility of neopterin levels for detecting active TB requires larger and lengthier studies.

In addition, lower neopterin levels were observed in our RA patients with borderline QFT-G conversion compared with those with non-borderline conversion, indicating borderline QFT-G conversion may be a laboratory variation and of limited relevance to active TB development [40]. None of our 12 borderline QFT-G converters developed active TB during the follow-up period. Therefore, serial QFT-G results should be interpreted cautiously due to their high variability, particularly for borderline QFT-G converters.

Interestingly, the levels of mitogen-stimulated levels of IFN-γ, IL-17A, and IL-13 were significantly lower in RA-TB patients compared with RA non-TB patients. In QFT-G assay, mitogen-stimulated IFN-γ response may reflect the host immunity. Other studies have shown that mitogen-stimulated IFN-γ levels were lower in patients with active TB in comparison to those without TB [34], and a reduced Th17 response was found in active TB patients [41]. The impaired immunity associated with active TB could be partly caused by suppressed cytokine production [42], reflected in our data showing no increase of proinflammatory cytokines (IFN-γ, IL-17A, and IL-13) in RA-TB patients, in contrast to their increases in RA non-TB patients (Fig 3).

There were some limitations in our study. Firstly, this study was hampered by the small number of RA patients with active TB, and thus it was not possible to illustrate the diagnostic value of neopterin levels. Secondly, the definition of QFT-G conversion applied in our study was arbitrary because there is no gold standard for that; nonetheless our definition was based on previous reports [15,31]. Thirdly, data on the exact timing of QFT-G conversion after biologic therapy are not currently available. Fourthly, because that borderline QFT-G (0.35–0.70 IU /ml) result might be positive or negative results, the borderline QFT-G results should be repeated. Finally, Taiwan is a TB-endemic area; our data cannot be meaningfully applied to countries with a low prevalence of TB. Therefore, our results require further confirmation by larger studies and more dynamic evaluation of neopterin levels to determine its clinical utility for detecting active TB in QFT-G converters.

**In conclusion,** our results in a real world setting indicate that elevated levels of neopterin are associated with TB risk in RA QFT-G converters undergoing biologic therapy. Close surveillance for active TB is mandatory when biologic-exposed RA patients have both QFT-G conversion and a rising neopterin level, particularly in TB-endemic areas.
